# Integration of Subthreshold and Suprathreshold Excitatory Barrages along the Somatodendritic Axis of Pyramidal Neurons

**DOI:** 10.1371/journal.pone.0033831

**Published:** 2012-03-23

**Authors:** Hysell V. Oviedo, Alex D. Reyes

**Affiliations:** 1 Cold Spring Harbor Lab, Cold Spring Harbor, New York, United States of America; 2 Center for Neural Science, New York University, New York, New York, United States of America; The Research Center of Neurobiology-Neurophysiology of Marseille, France

## Abstract

Neurons integrate inputs arriving in different cellular compartments to produce action potentials that are transmitted to other neurons. Because of the voltage- and time-dependent conductances in the dendrites and soma, summation of synaptic inputs is complex. To examine summation of membrane potentials and firing rates, we performed whole-cell recordings from layer 5 cortical pyramidal neurons in acute slices of the rat's somatosensory cortex. We delivered subthreshold and suprathreshold stimuli at the soma and several sites on the apical dendrite, and injected inputs that mimic synaptic barrages at individual or distributed sites. We found that summation of subthreshold potentials differed from that of firing rates. Subthreshold summation was linear when barrages were small but became supralinear as barrages increased. When neurons were discharging repetitively the rules were more diverse. At the soma and proximal apical dendrite summation of the evoked firing rates was predominantly sublinear whereas in the distal dendrite summation ranged from supralinear to sublinear. In addition, the integration of inputs delivered at a single location differed from that of distributed inputs only for suprathreshold responses. These results indicate that convergent inputs onto the apical dendrite and soma do not simply summate linearly, as suggested previously, and that distinct presynaptic afferents that target specific sites on the dendritic tree may perform unique sets of computations.

## Introduction

Integration of synaptic inputs depends on the various conductances in the dendrites, which are activated at different voltage ranges and are expressed at different densities along the somatodendritic axis [1]; [2]; [3]. Substantial progress has been made towards understanding how these conductances transduce synaptic inputs into neuronal firing in the dendrites [4]; [5]. More recent studies have shed light on integration along individual dendritic branchlets in neocortical pyramidal neurons by stimulating individual spines [6]. A combination of voltage gated conductances and asymmetric dendritic geometry produce an integration gradient along the proximal-distal dendritic axis with heterogeneous integration rules. Less understood are the rules for the integration of convergent inputs along the somatodendritic axis. A survey of previous studies on synaptic integration reveals contradicting results. In the neocortex, unitary excitatory postsynaptic potentials (EPSPs) evoked in postsynaptic pyramidal cells and interneurons following simultaneous stimulation of two presynaptic cells summed linearly when synaptic contacts were on separate branches and sublinearly when contacts were close [7]. In other studies, however, stimulus-evoked EPSPs summed supralinearly [8]; [9]; [10], suggesting activation of voltage-gated conductances that boost synaptic inputs [11]; [12]; [13]; [14].

Part of the discrepancy may arise from the fact that activation of conductances varies with the magnitude, location and timing of the inputs [15]; [16]; [1]; [7]. Hence, whether or not EPSPs summate linearly likely depends on the specific stimulus protocol. Brief EPSPs predominantly recruit fast conductances that are activated near resting potential, while sustained stimuli can also activate conductances with slow kinetics [17]; [1]. Further, spatially distributed inputs cannot activate voltage-gated conductances as effectively as closely-spaced inputs [4], [18].

Previous studies on the apical dendrite of layer 5 pyramidal neurons have examined the impact of stimulating different compartments on the input/output relationship [19]; [20]–[21]. These studies show that neuronal responses and temporal integration window vary depending on the location of the stimulus. It remains unclear how these heterogeneous, local input/output transformations along the somatodendritic axis converge to shape the final integrative properties of neurons. In a previous study [19], we characterized changes in the boosting of inputs injected at the soma and several dendritic compartments, as well as changes in the firing dynamics. In the present study we extend our previous findings by examining whether changes in the input/output relation translate into changes in the integrative properties at the soma and dendritic compartments.

In this study we examined the summation of subthreshold and suprathreshold responses by injecting inputs that mimic synaptic barrages at the soma and apical dendrites of layer 5 pyramidal neurons. The barrages were injected under current and dynamic clamp at individual and distributed sites along the somatodendritic axis. We found that subthreshold potentials summed linearly and became supralinear as the synaptic barrage increased. When inputs were injected at individual sites the degree of supralinear summation increased along the somatodendritic axis, whereas distributed inputs removed this spatial dependence. Summation in the suprathreshold range (where the barrages evoked repetitive firing) depended on the location of the inputs. At the soma and proximal dendrite, summation of firing rates became more sublinear as the synaptic barrages increased. In contrast, summation in the distal dendrites was initially supralinear and became sublinear with increasing input. These results suggest that various voltage-dependent conductances add a rich and complex set of integration rules that vary according to the magnitude, location and distribution of the inputs.

## Methods

### Ethics statement

Surgical and slicing techniques were as described previously [22] and followed guidelines set forth by the Animal Welfare Committee of NYU (animal assurance number 3317-01, Office of Veterinary Resources protocol number: 02-1154). Wistar rats (3–5 weeks old) were anesthetized with halothane and decapitated. One hemisphere of the brain was excised, glued to a slicing chamber, and immersed in ice cold, oxygenated artificial cerebrospinal fluid (ACSF) containing in mM: 125 NaCl, 2.5 KCl, 25 glucose, 25 NaHCO_3_, 1.25 NaH_2_PO_4_, 2 CaCl_2_, and 1 MgCl_2_. A vibratome slicer was used to make parasaggital (300 µm thick) slices cut at a 15° angle from the horizontal plane. The slices were stored in a holding chamber maintained at 34°C for 1 hour and at room temperature thereafter. Individual slices were transferred to a recording chamber mounted on an upright microscope and perfused with ACSF heated to 33–34°C. Layer 5 pyramidal neurons in somatosensory cortex were visualized and identified using infrared, differential interference contrast videomicroscopy. Whole-cell current clamp recordings were performed using borosilicate microelectrodes pulled to a diameter of 2 µm for somatic recordings and 1 µm for dendritic recordings. Electrodes had D.C. resistances of 5–20 MΩ when filled with (in mM): 100 K-gluconate, 20 KCl, 4 MgATP, 10 phosphocreatine, 0.3 GTP, and 10 HEPES. Voltage and current signals were filtered at 10 kHz using Cornerstone BVC-700 amplifiers (Dagan Corporation, Minneapolis, ME) and digitized at 2–10 kHz. Stimulus delivery, data acquisition and analyses were implemented in IGOR (Wavemetrics, Lake Oswego, OR).

Neurons were stimulated with inputs that mimic the composite synaptic current generated by the firing of a population of presynaptic excitatory neurons (see [14] for details). A computer program simulated the firing of a specified number of presynaptic cells (n) each firing at a specified rate (f_pre_). Therefore, the total incoming rate of excitatory postsynaptic current (EPSC) is equal to n* f_pre_. Jitter was added to the interspike intervals (ISIs) of each spike train such that the ISIs were distributed normally about a mean interval with a standard deviation of ±10% of the ISI. The start times of the spike trains were uniformly distributed within 1 ISI so that the simulated spike trains were uncorrelated.

Each time a simulated presynaptic cell fired an action potential, a single EPSC was calculated. The time course of each EPSC was described by 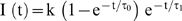 where k determines the amplitude of the synaptic input and τ_0_ and τ_1_ are time constants describing the rise and fall times of the postsynaptic current. When injected into a cell, a transient voltage deflection was evoked. The amplitude and time course were adjusted to match those of unitary EPSPs measured with paired recordings (amplitudes, 0.4–1.0 mV; [23]). Stimulus driven activity in the rat barrel cortex can range from 3 to 40 Hz (non-bursting rate; [24]) and the number of presynaptic cells that can drive a layer 5 pyramidal neuron can range from 30 to over 100 [25]. Therefore, we chose to stimulate each neuronal compartment with EPSC rates of up to 1–4 kHz.

**Figure 1:**
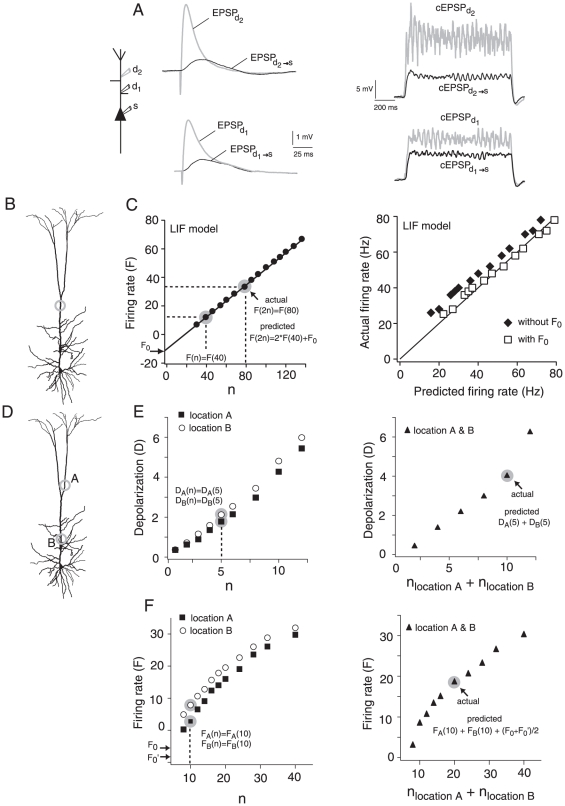
Matching EPSPs and calculating the linearly-predicted summation of inputs. A, left, EPSPs recorded at the sites of injection (EPSP_d1_, EPSP_d2_) and at the soma (EPSP_d1→s_, EPSP_d2→s_). Electrodes at d_1_ and d_2_ were placed 150 µm and 334 µm, respectively, from the soma. Right, Depolarizations (termed composite EPSPs or cEPSPs) recorded at the dendrite (cEPSP_d1_, cEPSP_d2_) and at the soma (cEPSP_d1→s_, cEPSP_d2→s_) when EPSC barrages (rate = 0.24 kHz) were injected at d_1_ or d_2_. B, Injecting the inputs at a single location to test if doubling the response evoked by n inputs equals the response to injecting 2n. C, The linear prediction of the firing rate incorporates the contribution of the inputs needed to reach threshold. Left, LIF model: the y-intercept (arrow) of the F vs. n relation was used as the correction factor (F_0_) for the linear prediction. Right, LIF model: plot of the actual and predicted firing rate with (□) and without (⧫) compensating for the inputs needed for threshold. D, Spatial summation: the response to inputs injected simultaneously at two separate dendritic locations (A and B) was compared to the sum of the inputs delivered individually. The total number of inputs injected at each location individually and simultaneously was identical. E, left, For spatial summation in the subthreshold range, the linear prediction was calculated by adding the depolarization measured by injecting n inputs (e.g. n = 5, points highlighted in gray) at each dendritic location individually. Right, The actual sum of the inputs was obtained by simultaneous injection of n inputs at each location (i.e. n_locationA_+n_locationB_ = 10 in data point highlighted in gray). F, left, For suprathreshold spatial summation, the predicted firing rate was calculated by adding the firing rates obtained by injecting n inputs (e.g. n = 10, points highlighted in gray) at each location individually. The correction for voltage threshold was calculated by taking the average of the y-intercepts (F_0_′, F_0_) of the F vs. n relation for each location. Right, The actual sum of the inputs was obtained by simultaneous injection of n inputs at each location (i.e. n_locationA_+n_locationB_ = 20, in data point highlighted in gray).

We matched the time course and amplitude of the dendritically evoked EPSPs recorded at the soma to facilitate the comparison of the responses obtained with injection of inputs at any location along the dendrite. Further, the focus of this study was to measure differences in the somatically recorded depolarization/firing rate produced by injecting the simulated inputs along the somatodendritic axis. Therefore, we wanted to ensure that the net current reaching the action potential initiation region was equal regardless of the location of the input. Briefly, for every cell the 3 free parameters (amplitude and two time constants) were adjusted until the average somatically recorded EPSPs evoked by current injection at all somatodendritic locations matched in amplitude and time course (Figure 1A, left). The amplitude, τ_0_ and τ_1_ of the EPSCs injected had the following parameters (in units of picoAmps and milliseconds, respectively): distal dendrite (0.08, 0.15, 2.5), proximal-middle dendrite (0.04, 1, 2.5), and soma (0.02, 2, 3).

To mimic a barrage of EPSCs, the unitary EPSCs were convolved with the spike trains of the simulated presynaptic cells [26]; [14]. The current trains from all the presynaptic cells were summed, converted to an analog signal, and injected into the cell via the amplifier and recording electrode. Stimuli were 1.2 seconds long and delivered at greater than 3-second intervals to ensure that the cells reached resting conditions between each stimulus. For every cell and for every neuronal compartment tested, we injected inputs to obtain the input-output transformation at both the subthreshold and suprathreshold level. We averaged about 10 trials per EPSC rate tested.

Some recordings were performed under dynamic clamp, a voltage-controlled current clamp that uses an analog multiplier to calculate and inject the current that would be produced by conductance changes [27]; [28]. This analog multiplier can update the current almost instantaneously as the voltage changes. We further reduced potential sampling errors by implementing the dynamic clamp with 2 electrodes to independently sample the membrane voltage and inject the current [29]. Excitatory currents were calculated as I_syn_ = g_syn_ (E_rev_−V), where g_syn_ is the computer-controlled synaptic conductance generated from the simulated presynaptic spike trains, E_rev_ is the reversal potential of the synaptic conductance (0 mV for excitatory inputs), and V is the membrane potential. Note that because the dynamic clamp is analog and not software driven the calculation of I_syn_ is effectively instantaneous (less than the membrane time constant of neurons), and not subject to potential aliasing effects.

Input barrages were injected at individual locations along the somatodendritic axis and simultaneously at several sites. We compared the observed (or actual) properties of integration with a predicted sum of the inputs that assumes linear summation.

### Calculating predicted linear summation

In the first part of this study we examined summation of progressively increasing inputs injected at different cellular compartments (Fig. 1A and D). In the subthreshold regime, the *actual* summation of potentials (V) resulting from a specified number of inputs (n) was compared to the *predicted* linear summation, which was calculated by doubling the depolarization measured with half the number of inputs (n/2). Hence, if summation is linear, V (n) = V (n/2)+V (n/2). Similar calculations were performed in the suprathreshold range, with average firing rate replacing V. Because of the presence of threshold, the number of inputs needed to raise the membrane potential to threshold (n_Th_) must be taken into account. To understand why this is necessary, consider the predicted linear summation of the firing rate in a leaky integrate-and-fire (LIF) model. Summation of firing rates in LIFs should be linear. Figure 1C shows a plot of firing rate vs. n (left) and a plot of actual vs. predicted response (right). Summation without the correction for threshold appears supralinear (⧫). Unlike subthreshold responses, the plot of firing rate vs. n does not pass through the origin (compare Fig. 1C and E, left). One way to factor in the contribution of n_Th_ is to do a linear fit through the linear portion of the firing rate vs. n relation and add the y-intercept (F_0_) to the linear prediction of the firing rate. With this correction (F_0_) the summation of the firing rate in the LIF model becomes linear (Fig. 1C, right panel, □).

The second part of this study examines summation of inputs delivered at individual locations or at two sites simultaneously on the apical dendrite (Fig. 1D). The responses obtained by stimulating each site individually (Fig. 1D, circles) were added to obtain the predicted linear sum. This was then compared to the actual response evoked when the inputs to each site were injected simultaneously. In the subthreshold range, the depolarization evoked by injecting the inputs simultaneously at two different dendritic locations (Fig. 1E right) was compared to the sum of the depolarization obtained by stimulating each location individually (Fig. 1E left). For the firing rate, we calculated the linear prediction by stimulating each site individually and adding the responses to the average of the y-intercepts (F_0_, F_0_′) of each firing rate curve (Fig. 1F left). This linear prediction was compared to the firing rate obtained with the simultaneous injection of the inputs (Fig. 1F, right panel).

### Blockade of Persistent sodium current

In some experiments, we examined the effects of blocking the persistent sodium current, I_NaP_, on the summation properties (see below). Riluzole (Sigma, USA) was dissolved in ACSF to a concentration of 10 µM [30]. Riluzole is a more specific blocker of I_NaP_ than TTX, especially at this low concentration, but it can also block the fast sodium current at higher concentrations [31].

To compare summation under control and block conditions, a linearity ratio was calculated (LR = actual response divided by the predicted response). The difference between these ratios was calculated and normalized by the control ratio ((LR_control_−LR_block_)/LR_control_ *100). For each EPSC rate the difference in linearity was averaged across cells and plotted ± S.D. A similar calculation of the difference in linearity was used for comparing summation of inputs injected under current and dynamic clamp.

### Statistical analyses

To compare integration between the three different neuronal compartments examined (soma, proximal-middle, and distal), we used one-way ANOVA corrected for multiple comparison (Tukey-Kramer difference criterion). We used linear regression to fit the relationship between predicted vs. actual responses to obtain the slope and y-intercept for individual compartments in each cell. These parameters were then used for statistical analyses. The same analysis procedures were used for subthreshold and suprathreshold responses. For comparisons of paired data, we used a standard paired t-test.

## Results

We performed simultaneous whole-cell recordings at the soma, at proximal to middle locations in the apical dendrite (200 to 400 µm from the soma), and at distal locations (400 to 600 µm). Computer-generated inputs that mimic the composite synaptic current generated by a population of presynaptic excitatory neurons firing repetitively and asynchronously were delivered to the different compartments [14]; [32]. The main advantages of using input barrages to examine integration rather than standard current step injection are that: 1) summation of individual postsynaptic potentials (PSPs) can be quantified more directly; 2) background noise is inherently present; 3) the timing of PSPs can be controlled [14]; and 4) dynamic clamp can be applied eventually to mixed excitatory and inhibitory inputs (not examined here). To simplify comparisons among the different sites, the amplitudes and time constants of the unitary EPSCs that make up the barrages were adjusted so that the resultant voltage deflections recorded at the soma were identical to each other and recorded unitary EPSPs [33]; [23]. By changing the rate of EPSCs, the magnitude of the barrages can be adjusted so as to produce either subthreshold depolarizations or repetitive firing.

### Integration of inputs injected at the soma

**Figure 2:**
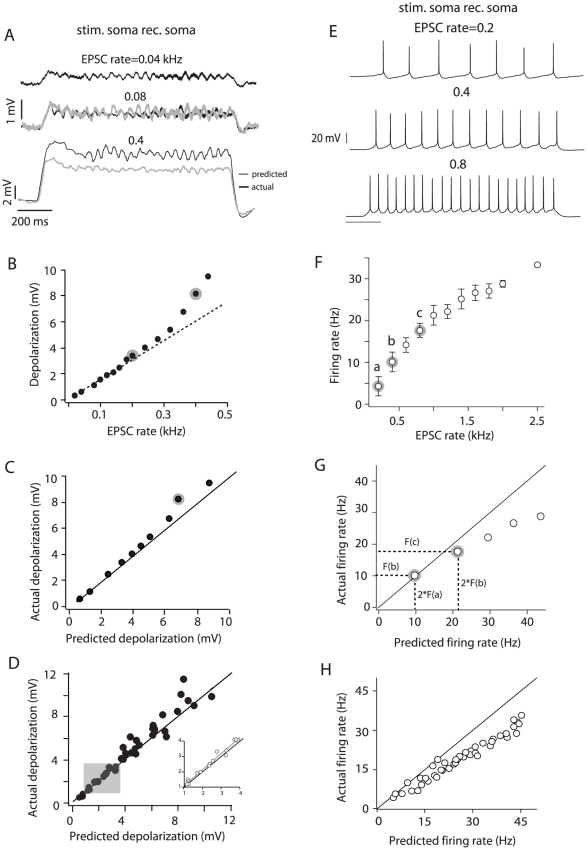
Integration at the soma. A, top voltage trace is the average depolarization obtained by injecting an input barrage with an EPSC rate of 0.04 kHz. This response was doubled to calculate the predicted depolarization when the input rate is doubled (0.08 kHz). The predicted response is overlaid with the actual response (gray and black middle traces, respectively). The bottom traces compare the actual and predicted responses at a higher input rate. B, Depolarization vs. input rate relation for the cell shown in A. The responses to 0.2 and 0.4 kHz (black bottom trace in A) are highlighted in gray. The dashed line is a linear fit through the first 7 data points. C, Actual vs. predicted depolarization for the cell shown in A and B. The point highlighted in gray corresponds to the actual depolarization measured at an input rate of 0.4 kHz and the prediction from doubling the response to 0.2 kHz (points highlighted in B). D, Population plot of the actual vs. predicted depolarization (n = 19). Shaded area corresponds to expanded data points (insert) and highlights responses between 2–4 mV. E, Summation of suprathreshold inputs at the soma (different cell from that shown in panels A–C). F, Firing rate vs. EPSC rate plot (± S.D.) for the cell shown in E. The firing rates marked a, b, c correspond to the spike trains in A, respectively. G, Actual vs. predicted firing rate for the cell shown E and F. The points highlighted correspond to the actual and predicted firing rates for points b and c in F. H, Population plot (n = 15) for suprathreshold integration at the soma. The solid black line in some plots represents the unity slope.

When we injected small input barrages (low EPSC rates), the resultant subthreshold depolarizations summed linearly at the soma. For example, the average membrane potential produced at an EPSC rate of 0.08 kHz was equal to doubling the response at 0.04 kHz (Fig. 2A, middle traces). At higher EPSC rates, summation became supralinear and the actual responses at 0.4 kHz (Fig. 2A, bottom traces) exceeded that predicted by doubling the response at 0.2 kHz (not shown). A plot of average depolarization vs. EPSC rate (Fig. 2B) shows that deviation from linearity starts gradually at an EPSC rate of approximately 0.2 kHz for this cell. The points highlighted in Fig. 2B and C show the substantial departure from linearity at more depolarized potentials. A plot of actual vs. predicted depolarization (Fig. 2C (one cell), D (*n* = 19)) shows that summation was significantly supralinear even at potentials between 2–4 mV (Fig. 2D gray box and expanded points; *p* = 0.012; *n* = 5, paired *t*-test).

To examine summation in the suprathreshold range, the EPSC rate was increased until the cell fired repetitively (Fig. 2E). To test for linearity, the *actual* average firing rate evoked at a given EPSC rate was compared to that *predicted* by doubling the firing rate obtained at half the EPSC rate (after adding a constant to account for the presence of a voltage threshold; see Methods). Although we occasionally observed linear summation at the soma (Fig. 2F and G, points ***a***–***b***), summation was mostly sublinear (Fig. 2F and G, points ***b***–***c***). A plot of actual vs. predicted firing rate for the population data shows that most of the points were below the unity slope line (Fig. 2H, *p*<0.0001, *n* = 19; paired *t*-test). Sublinear summation of firing rates likely results from the recruitment of voltage-gated conductances that underlie firing rate adaptation [34]; [35].

To summarize, the rules for synaptic integration at the soma varied with the input rate of the barrages. In the subthreshold range, summation of the membrane potential was linear for very low input rates, but became supralinear with increasing rates. In the suprathreshold range summation of firing rate was mostly sublinear.

### Integration of inputs in the apical dendrite

**Figure 3:**
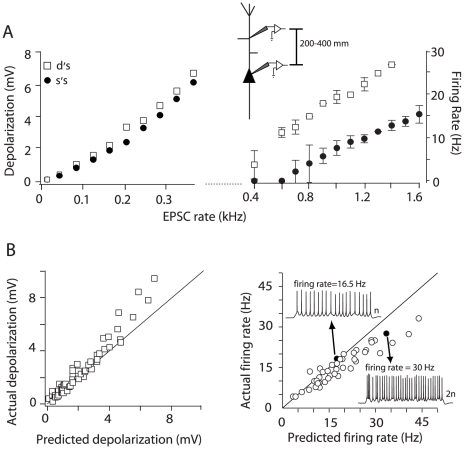
Integration of inputs injected at proximal-middle dendritic sites. A, left, Depolarization vs. EPSC rate relation for inputs injected at the soma (•) and dendrite (□, 270 µm away from soma) of the same neuron. Right, Suprathreshold continuation of the input-output relation. The inset shows the range of dendritic distances tested for integration of proximal-middle inputs. B, Population plots of the actual vs. predicted summation of subthreshold (left) and suprathreshold inputs (right) injected at proximal-middle sites (n = 15). Right, Black data points (and the corresponding spike trains) are the responses to a doubling of the input rate. The solid black line in some plots represents the unity slope.

The subthreshold and suprathreshold responses evoked with barrages injected at the apical dendrite were previously shown to be greater than those evoked with injection at the soma [14], [19]. This boosting was due to activation of dendritic Na^+^ conductances. To examine the effects of dendritic conductances on integration, we injected the input barrages at the apical dendrite, approximately 200–400 µm from the soma (henceforth termed proximal-middle sites) and compared the responses evoked with barrages injected at the soma (Fig. 3A, inset). The subthreshold depolarizations were similar to those at the soma at low EPSC rates but diverged at higher rates as the depolarization approached threshold (Fig. 3A, left). The differences in the responses to somatically and dendritically injected barrages were magnified in the suprathreshold range, where the firing rate evoked at the dendrite was significantly greater than that evoked at the soma (Fig. 3A, right).

Despite the boosting effects, the changes in summation properties were qualitatively similar at the soma and proximal-middle dendrite. As in the soma (Fig. 2C and D), subthreshold potentials summated linearly but became supralinear at higher EPSC rates (Fig. 3A and B, left). Although summation in the suprathreshold range was predominantly sublinear (Fig. 3B, right), at firing rates below 20 Hz the difference between the predicted and actual firing rates was not significant (*p* = 0.08, *n* = 10; paired *t*-test).

**Figure 4:**
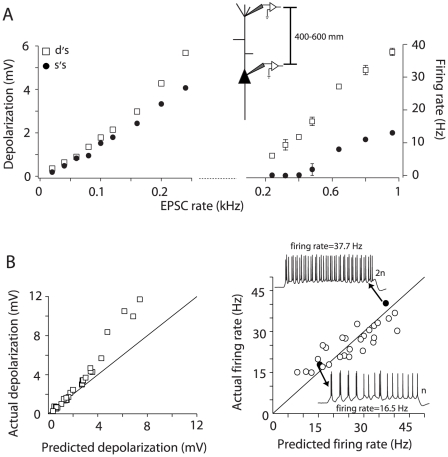
Integration of inputs injected at distal dendritic sites. A, left, Depolarization vs. input rate relation for inputs injected at the soma (•) and dendrite (□, 500 µm from the soma) of the same neuron. Right, suprathreshold continuation of the input-output relation. B, Population scatter plot of the actual vs. predicted summation of subthreshold (left) and suprathreshold (right) inputs injected at distal sites (n = 8). Right, Black data points (and the corresponding spike trains) are the responses to a doubling of the input rate. The solid black line in some plots represents the unity slope.

We also injected barrages at more distal sites, 400–600 µm from the soma. Injection of subthreshold barrages produced responses that were similar to those with injection at the proximal dendrite: the membrane potentials were boosted compared to somatic injection (Fig. 4A, left) and summation switched from linear to supralinear with increasing EPSC rates (Fig. 4B, left). The most significant difference occurred in the suprathreshold range (Fig. 4A, right) where summation of firing rates was initially supralinear (points above the diagonal, Fig. 4B, right) and became linear to sublinear at higher firing rates. Over the entire range of responses tested the difference between the predicted and actual firing rate was not significant (*p* = 0.39, *n* = 7; paired *t*-test).

**Figure 5:**
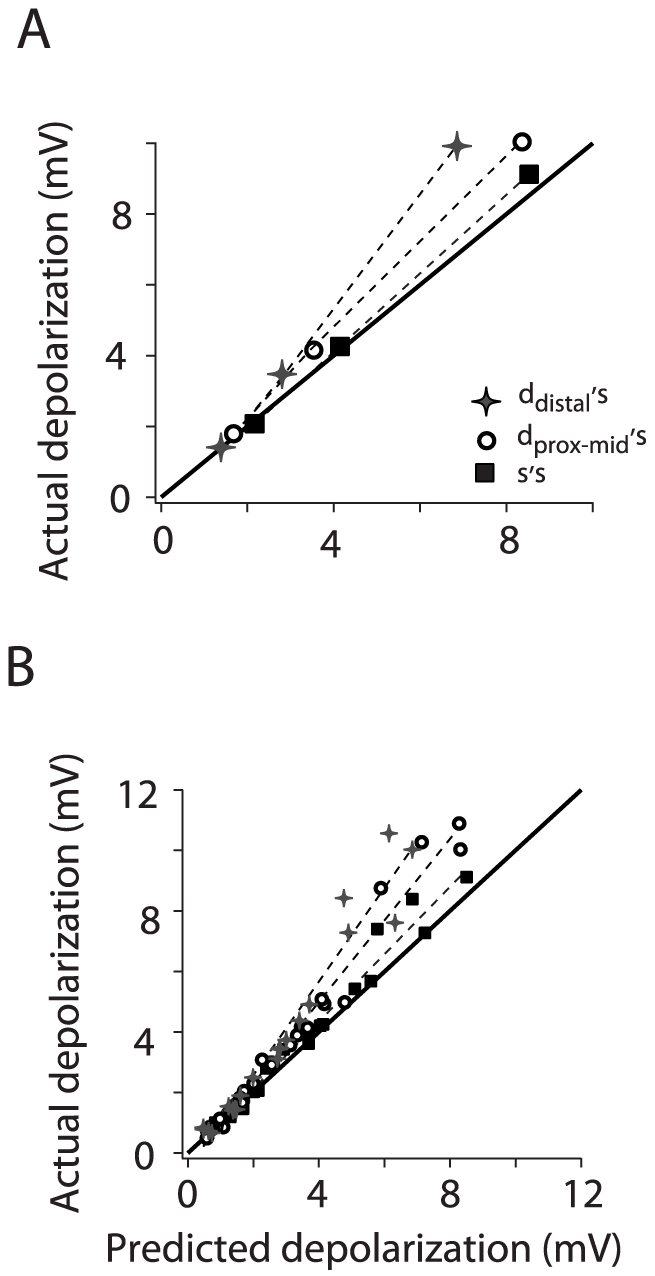
Summary of subthreshold integration along the somatodendritic axis. A, Actual vs. predicted depolarization for one cell when the inputs were injected at the soma (square), proximal-middle (230 µm, circle) and distal dendritic sites (500 µm, star). The same input rates were tested at all locations (0.04, 0.08, and 0.16 kHz). B, Population scatter plot of the actual vs. predicted depolarization at the soma, proximal-middle and distal dendrite. The dash lines are linear fits through the data points. For each cell plotted (n = 7) summation was tested at all three locations. The black line in both panels represents the unity slope.

A closer look at the summation of subthreshold responses revealed that it was quantitatively different at all the neuronal compartments tested. Figure 5A compares summation for one cell when inputs were injected at the soma, proximal-middle and distal dendrite. For the same input rates, doubling their magnitude at each location caused summation to change from linear to supralinear. However, the degree of supralinearity was greater at more distal sites. To test whether the differences in supralinearity (i.e. changing slopes) between the different compartments was significant, we used one-way ANOVA corrected for multiple comparisons. We found that there was a significant difference in the slope of the predicted vs. actual responses between the soma and all dendritic compartments, but not between dendritic compartments (F = 12, P<0.001, n = 7; Fig. 5B).

Summation of suprathreshold responses was also quantitatively different in all the compartments tested. The parameters used to test differences in integration were the y-intercept and slope of the linear regression of the actual vs. predicted firing rate relation for each cell. The difference in slope was significant, in particular between the soma and distal dendritic sites (one-way ANOVA, F = 10.3, P<0.001, n = 10). The difference in the y-intercepts was also significant, in particular between the soma and distal sites, and between the dendritic compartments (one-way ANOVA, F = 5, P<0.01, n = 10). Comparing the spike trains evoked at the distal (Fig. 4B, right) and proximal-middle (Fig. 3B, right) sites underscores the quantitative differences in summation properties. For a given number of inputs (n) that evoke the same firing rate when injected at each location (Fig. 3B and 4B right, black circles), doubling the inputs (2n) led to sublinear integration at the proximal-middle site but not the distal dendrite.

### Effects of blocking I_NaP_

The persistent sodium current, I_NaP_, has been shown to amplify inputs to the dendrite [36]. To examine the contribution of I_NaP_ to summation, we bath-applied the specific blocker Riluzole while injecting barrages at proximal-middle dendritic sites. Blocking I_NaP_ had no effect on the resting membrane potential but increased the threshold for action potential generation (data not shown).

**Figure 6:**
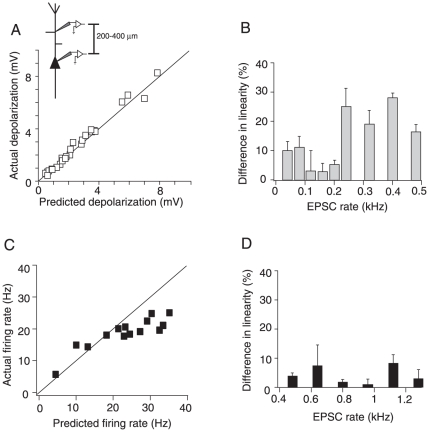
Effects of blocking I_NaP_ on the integration properties of proximal-middle dendritic sites. A, Population scatter plot (n = 5) of the actual vs. predicted depolarization in the presence of the I_NaP_ blocker Riluzole. Most points lie on the unity slope line. B, Percent difference in the linearity (± S.D.) of summation between control and I_NaP_ block conditions plotted against input rate for the population of cells shown in A. C, Actual vs. predicted firing rate (n = 5) in the presence of Riluzole. D, Percent difference in the linearity of the firing rate between control and I_NaP_ block conditions for the data shown in C.

With I_NaP_ blocked, summation of membrane potentials became linear at all input rates: the difference between the actual and predicted depolarization was not statistically significant (Fig. 6A; *p* = 0.112, *n* = 6; paired *t*-test). The percent change in linearity attributable to I_NaP_ ((LR_control_−LR_block_)/LR_control_ *100, see Methods) was positive, confirming that integration is supralinear prior to I_NaP_ block (Fig. 6B). The effect of I_NaP_ block was most apparent at EPSC rates greater than 0.2 kHz where summation deviated from linearity under control conditions (Fig. 2B).

Blocking I_NaP_ significantly decreased the dendritically evoked firing rate compared to control conditions (Fig. 6C and D, *p* = 0.018, *n* = 6; *t*-test). The percent change in linearity was positive, indicating that summation became more sublinear following block of I_NaP_ (Fig. 6D). However, the dependence of summation properties on input rate was qualitatively unaffected: summation became increasingly sublinear at higher firing rates (Fig. 6C).

### Effects of synaptic shunting on integration

**Figure 7:**
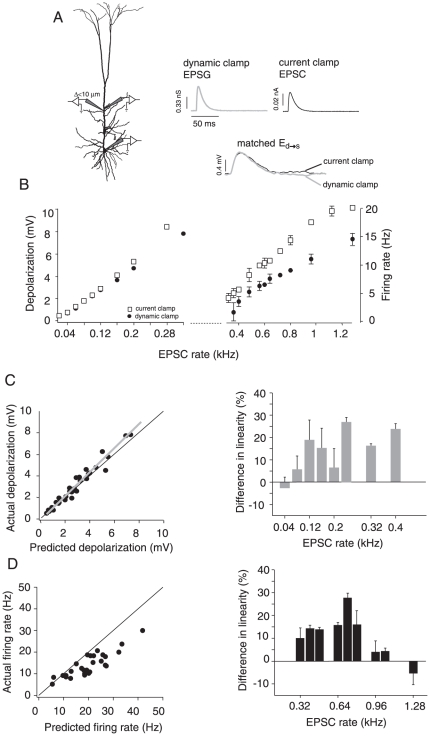
Comparison of integration properties under current and dynamic clamp. A, left, Schematic of the stimulus delivery and recording set-up under dynamic clamp. Two electrodes (<10 µm apart) were placed at the proximal-middle dendrite, and a third was placed at the soma to record the output. Right, The amplitudes of the unitary excitatory postsynaptic conductances (EPSGs) and EPSCs (top traces) injected at the dendrite were adjusted so that the resulting EPSPs at the soma (E_d→s_) were identical (overlaid on bottom traces). B, Subthreshold (left) and suprathreshold (right) input/output relations under dynamic (•) and current clamp (□) for one cell (injection site: 260 µm from soma). C, left, Population plot of subthreshold integration under dynamic clamp showing the actual vs. predicted depolarization (n = 8). The gray line is the linear fit through the data points. Right, percent difference in the linearity (± S.D.) of integration between current and dynamic clamp plotted against input rate for the population data shown on the left. D, left, Population data for suprathreshold integration under dynamic clamp (n = 8). Right, Plot of difference in linearity between current and dynamic clamp for the data shown in left panel. The solid black line in some plots represents the unity slope.

To simulate the changes in conductance caused by electrotonically close synaptic inputs, we injected the barrages under dynamic clamp (see Methods and [32]). Two electrodes spaced less than 10 µm apart (one for recording voltage and the other for injecting current) were placed in the proximal-middle segments (230 µm±50) and a third electrode placed in the soma (Fig. 7A, left). The EPSC amplitude injected under current clamp was adjusted so that the resulting EPSP recorded at the soma matched the EPSP evoked with the dynamic clamp (Fig. 7A, right). The responses evoked with the dynamic clamp were then compared to those evoked with current clamp. As expected, the responses evoked under dynamic clamp began to diverge from those evoked with current clamp as the input rate increased in both the subthreshold and suprathreshold range (Fig. 7B left and right, respectively). This is caused by the average membrane potential approaching the EPSP reversal potential (0 mV).

The summation properties obtained under dynamic clamp were similar to those obtained under current clamp. In the subthreshold regime, summation changed from linear to supralinear as input increased (Fig. 7C, left). However, summation was significantly more supralinear under current clamp (Fig. 7C, right; (LR_current_clamp_−LR_dynamic_clamp_)/LR_current_clamp_ *100; *p*<0.0001, *n* = 8; *t*-test). The opposite trend occurred with suprathreshold summation; the difference in linearity between current and dynamic clamp decreased as input rate increased (compare right panels in Fig. 7C and D). This may be due to the fact that the firing rates under both conditions asymptotically approached the maximally attainable firing rates. Nevertheless, suprathreshold summation was significantly more sublinear with shunting (Fig. 7D, left panel; *p* = 0.003, *n* = 8; *t*-test).

### Summation of spatially distributed inputs

**Figure 8:**
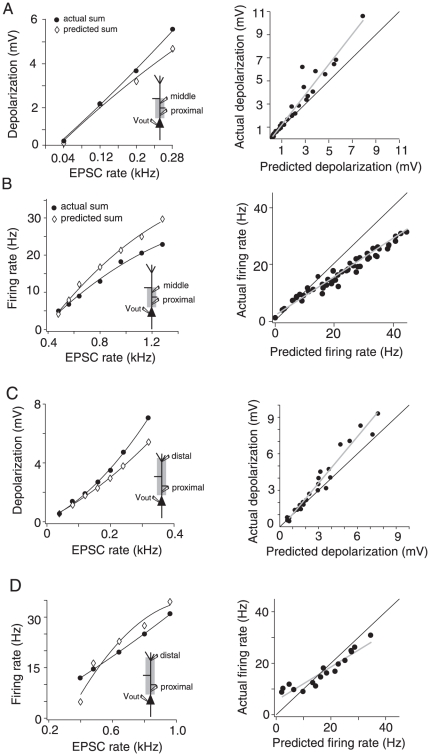
Integration of inputs distributed between proximal-middle (A and B) and proximal-distal (C and D) dendritic sites. A, Left, Actual and predicted depolarization vs. input rate for one cell stimulated at 115 µm and 300 µm from the soma. Right, Population plot (n = 15) of the actual vs. predicted spatial summation of subthreshold inputs injected at proximal-middle sites. B, Left, Actual and predicted firing rate vs. input rate relation for one cell stimulated at 115 µm and 300 µm from the soma. Right, Population plot (n = 15) of the actual vs. predicted spatial summation of suprathreshold inputs injected at proximal-middle sites. C, Left, Actual and predicted depolarization vs. input rate for one cell stimulated at 100 µm and 500 µm from the soma. Right, Population plot (n = 7) of the actual vs. predicted spatial summation of subthreshold inputs injected at proximal-distal sites. D, Left, Actual and predicted firing rate vs. input rate relation for one cell stimulated at 100 µm and 500 µm from the soma. Right, population plot (n = 7) of the actual vs. predicted spatial summation of suprathreshold inputs injected at proximal-distal sites. In all single cell examples (left column) black lines are polynomial fits through the data. In all population plots (right column) black lines are the unity slopes and gray lines are best linear fit through the data points.

The density of some conductances change along the apical dendrite (for reviews see [1] and [2]), suggesting that summation of spatially distributed inputs might be different from summation of clustered inputs. To investigate the summation rules of distributed inputs we performed triple whole-cell recordings at the soma and two sites on the apical dendrite. We injected the input barrages simultaneously at proximal (77 to 163 µm; mean: 109 µm±33.2) and middle (200 to 334 µm; mean: 270 µm±54), and proximal and distal (400 to 600 µm; mean: 500 µm±62) dendritic sites. Different realizations of the input current were delivered to each site. This may cause trial-to-trial variability in the responses, but not the average steady-state integration measured here. As was observed for injection at a single site, summation of subthreshold potentials was linear but became supralinear at higher EPSC rates (Fig. 8A and C). Summation of firing rates in the suprathreshold range was also qualitatively similar for both dual and single injections: at proximal-middle sites summation was mostly sublinear (Fig. 8B) and stimulating distal sites made summation supralinear at some firing rates (Fig. 8D).

Dual injection at the proximal and distal apical dendrite produced suprathreshold summation properties that resembled those observed with injection at the distal dendrite alone: summation was initially supralinear and became sublinear with increasing input rate (Fig. 8D, left). There was no significant difference between the predicted and actual firing rates in the pooled data (Fig. 8D, right, *p* = 0.75, *n* = 6; paired *t*-test). A comparison between the suprathreshold population data for proximal-middle and proximal-distal spatial integration shows that there was a significant difference between the two spatial distributions. The linear fits through the predicted and actual responses of individual cells revealed a significant difference between the y-intercepts (Fig. 8B and D, right panels, *p*<0.05, p = 0.0961, respectively; *n* = 8; paired *t*-test), but not the slopes. However, there was no significant difference in the slopes of the linear fits in the subthreshold spatial integration (Fig. 8A and C, right panels; *p* = 0.0968, *n* = 8; paired *t*-test).

## Discussion

In this study we examined integration of synaptic input at different compartments of layer 5 pyramidal cells. We extend previous studies of summation of synaptic potentials [37]; [8]; [9]; [10]; [7] by delivering synaptic barrages, which mimic the total synaptic input from a population of repetitively firing presynaptic cells. The barrages were adjusted so that the evoked responses ranged from subthreshold depolarization to suprathreshold firing. In the subthreshold range, summation was qualitatively similar at the different cellular compartments: summation was linear at low input rates but became increasingly supralinear as the input rate increased and the membrane potential approached the action potential threshold. The degree of supralinearity increased with distance from the soma when inputs were delivered at individual locations but did not change when the inputs were delivered simultaneously at separate compartments. In contrast, summation in the suprathreshold range changed both qualitatively and quantitatively with distance from the soma and with the spatial distribution of the inputs. Inputs delivered at distal sites activated voltage-dependent conductances that led to a combination of supralinear (for firing rates of up to 40 Hz), linear and sublinear summation. Further, the sublinearity of summation from distal stimulation was less pronounced than at the soma (i.e. at distal sites most sublinear firing rates stay close to the unity slope line; compare Fig. 2H and 4B right).

Injecting inputs under current clamp mimics the condition where synaptic inputs from electrotonically and spatially distant branches converge at a common site (e.g. at the dendritic recording sites). Whereas injecting the barrage under dynamic clamp simulates the case where inputs are close to each other on the same branch. Under this condition, there is mutual shunting of the inputs. We found that summation of electrotonically close inputs (achieved with the dynamic clamp) made integration more sublinear. Table 1 summarizes the summation properties for all the conditions tested. These compartment-dependent integration rules are likely to apply under *in vivo* conditions where there is a higher level of background synaptic activity. An *in vitro* study simulating in vivo high-conductance conditions found that the conductance load resulting from synaptic inputs is spatially compartmentalized allowing neuronal compartments to sum inputs independently [32].

**Table 1:** Summary of integration rules.

	SPATIALLY CLUSTERED			SPATIALLY DISTRIBUTED		
	soma	prox Mid	distal	PROX+MID	PROX+DIST	shunting
SUB	L→SP	L→SP	L→SP	L→SP	L→SP	L→SP
SUPRA	SB	L→SB	L	L→SB	L	SB

Rules of integration for inputs injected at a single location along the somatodendritic axis (SPATIALLY CLUSTERED), injected simultaneously (SPATIALLY DISTRIBUTED) and injected under dynamic clamp (SHUNTING). Rows correspond to subthreshold (SUB) and suprathreshold (SUPRA) integration. PROX MID = stimulation of proximal to middle sites, PROX+MID = simultaneous stimulation of proximal and middle sites, PROX+DIST = simultaneous stimulation of proximal and distal sites, L = linear, SP = supralinear, SB = sublinear.

Since the focus of our study was to measure differences in the somatic depolarization/firing rate produced by injecting the simulated inputs along the somatodendritic axis, we adjusted single EPSCs injected at all sites so that the resultant voltage deflections near the spike initiation region (measured with the somatic electrode), were nearly identical regardless of the injection site. In this way, any nonlinearity introduced by dendritic conductances at the sites of injection can be examined exclusively. Consequently, the amplitude of the injected inputs increased with distance from the soma. Such synaptic scaling, while present in CA1 pyramidal cells [38], [39], do not appear to be present in layer 5 pyramidal cells [40]. The results nevertheless apply because summation was examined relative to average depolarization or firing rate (e.g. Figs. 2F–G, 3A right and 3B right, 4A right and 4B right). Using a fixed-amplitude EPSC would simply mean using systematically higher EPSC rates with increasing distance from the soma to maintain the same level of depolarization or firing rate.

### Subthreshold integration

Amplification of subthreshold inputs is voltage dependent and is partially mediated by I_NaP_. TTX-sensitive Na^+^ conductances have been shown to mediate the boosting of dendritically evoked responses [41]; [11]; [14]. The persistent sodium conductance (I_NaP_) in the dendrite [36]; [42] has been shown to amplify the net current reaching the soma. Previous studies have shown that single EPSPs with amplitudes greater than 5 mV are amplified by axo-somatic Na^+^ channels [37]. In this study, amplification occurred at an even lower level of depolarization (<4 mV, Fig. 2D gray area) because the injected synaptic barrages produced tonic depolarization, which likely activated I_NaP_ to a greater degree. The boosting by I_Nap_ was attenuated in electrotonically close inputs due to shunting effects.

**Figure 9:**
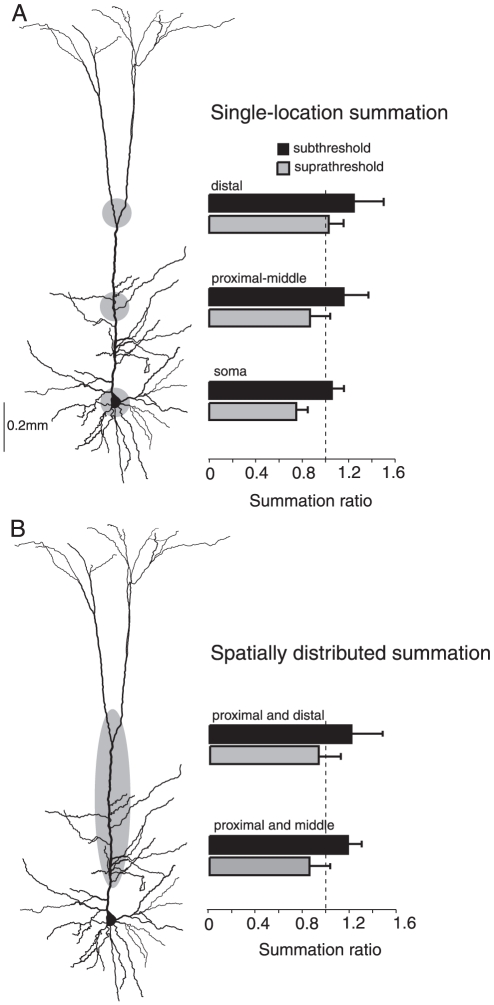
Summary of integration properties along the somatodendritic axis. A, Summary (n = 15) for inputs injected at individual locations (soma, proximal-middle and distal dendrite). For each location a summation ratio (actual response/expected response, ± S.D.) was calculated for subthreshold (black bars) and suprathreshold (gray bars) responses. The dotted line marks a summation ratio of 1 (actual response = expected response, indicating linear integration). B, Summary (n = 7) of integration of spatially distributed inputs.

In general, we find a distance dependent enhancement of subthreshold responses. Enhancement increases gradually at all locations and becomes more pronounced closer to the action potential threshold. This would explain the apparent discrepancy with a previous study [20] where enhancement was not observed with dendritic injections that produced somatic EPSPs of approximately 1 mV. At these low membrane potentials amplification is barely detectable (Fig. 2C). Inputs delivered at the distal dendrites summed more supralinearly than those delivered directly at the soma. This could be due to the higher density of voltage-dependent Ca^2+^ channels at distal sites of the apical dendrite ([43]; Fig. 9A). Ca^2+^ imaging of layer 5 pyramidal dendrites have demonstrated Ca^2+^ accumulation in the apical dendrite, with the highest concentration around the main bifurcation of the apical dendrite at 500 µm [44]; [43]. Dendritic Ca^2+^ transients can be activated even with small subthreshold potentials [45] and clustered input volleys at the apical dendrite would optimally activate these distal Ca^2+^ channels. Our results support this mechanism in that supralinear summation was attenuated when inputs were delivered at spatially distributed sites on the cell (compare Fig. 5 with 8A and C, right). Recent studies have shown the importance of NMDA dendritic conductances in the temporal discrimination of synaptic inputs [6]; [46]. Inputs that arrive in close temporal and spatial proximity can be enhanced via NMDA conductances. Although in our dynamic clamp experiments we did not mimic an NMDA component, we do expect this conductance to be another contributor to the enhancement of supralinear summation of inputs arriving synchronously.

### Suprathreshold integration

In a previous study [19] we found that several characteristics of suprathreshold activity depend on the somato-dendritic origin of the inputs and their strength. Inputs delivered to distal sites on the apical dendrite cause more burst firing and more variable interspike intervals than inputs delivered at the soma and proximal dendrite. The focus of the present study was on relative changes in the average firing rate, rather than the temporal dynamics of spiking. Previous studies have examined changes in the F/I relationship along the somatodendritic axis using noisy currents [21] [20]. Similar to our previous study [19] they have found that the most remarkable change in the F/I relationship of L5 pyramidal cells arises from the increase in gain and variability of firing when distal sites are stimulated. However, how changes in the F/I relationship translate into integrative properties has to be determined directly. For example, it is unclear from these studies whether the increase in gain from stimulation of the distal dendrite lead to sublinear, linear or supralinear summation, or a combination. Furthermore, our present study takes a comprehensive approach to characterize the integrative properties of each neuronal compartment directly from subthreshold to suprathreshold responses and by stimulating sites individually and simultaneously.

We found that for barrages delivered at the soma and proximal dendrite, firing rate increased sublinearly with input rate. Conductances underlying spike frequency adaptation likely limit the firing rate [34]; [35], causing the firing rates to asymptotically approach a maximum value. In the distal dendrites, firing rate increased more linearly than at proximal sites. Ca^2+^ channels in the distal dendrites affect summation in two ways. First, threshold input barrages delivered to the distal dendrites evoke more bursts than at perisomatic compartments [47]; [48]; [49]; [19]. And second, for larger input barrages Ca^2+^ mediated plateau potentials appear, reducing sublinear summation by triggering a combination of regular spikes and bursts (Fig. 4B, right, spike trains; [50]). Even when inputs were spatially distributed between proximal and distal sites, some burst firing was triggered in the low to mid firing range, and greater input rates evoked Ca^2+^-plateau events that led to greater burst firing at the soma [19], [21], [20]. Table 2 summarizes the spatial dependence of the integration rules.

**Table 2:** Spatial properties of integration.

	SPATIALLY CLUSTERED	SPATIALLY DISTRIBUTED
SUB	Increases distance dependence	No distance dependence
SUPRA	Increases distance dependence	Increases distance dependence

Summary of the changes in integration with the spatial distribution of the inputs. The spatially clustered column corresponds to input delivered at individual somatodendritic locations. The spatially distributed column corresponds to inputs delivered to two dendritic locations simultaneously.

### Functional implications

Integration of synaptic inputs in dendrites has been postulated to occur in 2 stages [16]. The first stage is composed of nonlinear ‘subunits’ that transform the summed synaptic inputs via a sigmoidal thresholding nonlinearity. This stage of computation is proposed to occur in the thin terminal branches of a dendritic tree. In the second stage, the outputs of the subunits from throughout the dendritic tree are summed at the apical dendrite and soma [16]. The integrative properties of these subunits have been examined using simultaneous synaptic stimulation of two sites on basal dendritic branches [4] and [18]. Stimulating sites less than 40 µm apart produced strong NMDA-dependent supralinear integration. However, integration was mostly linear if the stimulating electrodes were placed on different branches or more than 100 µm apart. Therefore, synaptically evoked boosting leading to supralinear integration mainly occurs for clustered inputs. The experimentally derived integration operation resembles the sigmoidal output function proposed by the first stage of the 2-layer model.

However, the 2-layer model does not account for all the complexities in the final stages of integration, such as the interactions between the regular spiking perisomatic output zone and the Ca^2+^-spiking distal dendrite [47] and NMDA-spiking basal and tuft dendrites [51]; [5]. To account for spiking in individual dendritic branches a 3-layer model has been proposed, where the outputs of the first layer (individual dendritic branches) feed into either a perisomatic or distal dendritic integration zones [52]; [53]; [54], [5]. Our results show that the final stage of integration (apical dendrite to axo-somatic compartment) does not sum inputs linearly and instead has a complex set of rules that change with neuronal activity (subthreshold vs. suprathreshold) and distribution of inputs.

One of the surprising findings of this study is that distributed inputs can add supralinearly on the apical dendrite. However, distributed inputs do eliminate the distance dependence of subthreshold summation, possibly due to the reduced activation of local Ca^2+^ conductances (Fig. 9B, black bars). Consequently, summation is likely to be invariant when the cell receives synaptic activity throughout the apical trunk. On the other hand, suprathreshold integration of distributed inputs becomes more linear as the inputs approach the low-threshold zone for Ca^2+^ events (Fig. 9A and B, gray bars). Therefore, the dendritic events triggered by spiking activity make suprathreshold integration in layer 5 pyramidal neurons distance dependent. There is evidence for both clustered and distributed organization of synaptic input from different pathways targeting the dendritic tree of L5 pyramidal neurons [55]. Pathways relaying thalamocortical information target the entire dendritic arborization of L5 pyramids, whereas cortico-cortical pathways cluster their input in perisomatic and distal dendrites. This suggests that there is differential processing (e.g. integration rules) of different pathways based on their location and distribution.

## References

[1] Reyes A (2001). Influence of dendritic conductances on the input-output properties of neurons.. Annu Rev Neurosci.

[2] Gulledge AT, Kampa BM, Stuart GJ (2005). Synaptic integration in dendritic trees.. J Neurobiol.

[3] Branco T, Hausser M (2010). The single dendritic branch as a fundamental functional unit in the nervous system.. Curr Opin Neurobiol.

[4] Polsky A, Mel BW, Schiller J (2004). Computational subunits in thin dendrites of pyramidal cells.. Nat Neurosci.

[5] Larkum ME, Nevian T, Sandler M, Polsky A, Schiller J (2009). Synaptic integration in tuft dendrites of layer 5 pyramidal neurons: a new unifying principle.. Science.

[6] Branco T, Clark BA, Hausser M (2010). Dendritic discrimination of temporal input sequences in cortical neurons.. Science.

[7] Tamas G, Szabadics J, Somogyi P (2002). Cell type- and subcellular position-dependent summation of unitary postsynaptic potentials in neocortical neurons.. J Neurosci.

[8] Schwindt PC, Crill WE (1998). Synaptically evoked dendritic action potentials in rat neocortical pyramidal neurons.. J Neurophysiol.

[9] Margulis M, Tang CM (1998). Temporal integration can readily switch between sublinear and supralinear summation.. J Neurophysiol.

[10] Nettleton JS, Spain WJ (2000). Linear to supralinear summation of AMPA-mediated EPSPs in neocortical pyramidal neurons.. J Neurophysiol.

[11] Lipowsky R, Gillessen T, Alzheimer C (1996). Dendritic Na+ channels amplify EPSPs in hippocampal CA1 pyramidal cells.. J Neurophysiol.

[12] Gillessen T, Alzheimer C (1997). Amplification of EPSPs by low Ni(2+)- and amiloride-sensitive Ca2+ channels in apical dendrites of rat CA1 pyramidal neurons.. J Neurophysiol.

[13] Gonzalez-Burgos G, Barrionuevo G (2001). Voltage-gated sodium channels shape subthreshold EPSPs in layer 5 pyramidal neurons from rat prefrontal cortex.. J Neurophysiol.

[14] Oviedo H, Reyes AD (2002). Boosting of neuronal firing evoked with asynchronous and synchronous inputs to the dendrite.. Nat Neurosci.

[15] Gasparini S, Magee JC (2006). State-dependent dendritic computation in hippocampal CA1 pyramidal neurons.. J Neurosci.

[16] Poirazi P, Brannon T, Mel BW (2003). Pyramidal neuron as two-layer neural network.. Neuron.

[17] Golding NL, Jung HY, Mickus T, Spruston N (1999). Dendritic calcium spike initiation and repolarization are controlled by distinct potassium channel subtypes in CA1 pyramidal neurons.. J Neurosci.

[18] Nevian T, Larkum ME, Polsky A, Schiller J (2007). Properties of basal dendrites of layer 5 pyramidal neurons: a direct patch-clamp recording study.. Nat Neurosci.

[19] Oviedo H, Reyes AD (2005). Variation of input-output properties along the somatodendritic axis of pyramidal neurons.. J Neurosci.

[20] Williams SR (2005). Encoding and decoding of dendritic excitation during active states in pyramidal neurons.. J Neurosci.

[21] Larkum ME, Senn W, Luscher HR (2004). Top-down Dendritic Input Increases the Gain of Layer 5 Pyramidal Neurons.. Cereb Cortex.

[22] Stuart GJ, Sakmann B (1994). Active propagation of somatic action potentials into neocortical pyramidal cell dendrites.. Nature.

[23] Reyes A, Sakmann B (1999). Developmental switch in the short-term modification of unitary EPSPs evoked in layer 2/3 and layer 5 pyramidal neurons of rat neocortex.. J Neurosci.

[24] Simons DJ (1978). Response properties of vibrissa units in rat SI somatosensory neocortex.. J Neurophysiol.

[25] Dodt HU, Schierloh A, Eder M, Zieglgansberger W (2003). Circuitry of rat barrel cortex investigated by infrared-guided laser stimulation.. Neuroreport.

[26] Reyes AD, Rubel EW, Spain WJ (1996). In vitro analysis of optimal stimuli for phase-locking and time-delayed modulation of firing in avian nucleus laminaris neurons.. J Neurosci.

[27] Sharp AA, O'Neil MB, Abbott LF, Marder E (1993). Dynamic clamp: computer-generated conductances in real neurons.. J Neurophysiol.

[28] Robinson HP, Kawai N (1993). Injection of digitally synthesized synaptic conductance transients to measure the integrative properties of neurons.. J Neurosci Methods.

[29] Chance FS, Abbott LF, Reyes AD (2002). Gain modulation from background synaptic input.. Neuron.

[30] Spadoni F, Hainsworth AH, Mercuri NB, Caputi L, Martella G (2002). Lamotrigine derivatives and riluzole inhibit INa,P in cortical neurons.. Neuroreport.

[31] Urbani A, Belluzzi O (2000). Riluzole inhibits the persistent sodium current in mammalian CNS neurons.. Eur J Neurosci.

[32] Williams SR (2004). Spatial compartmentalization and functional impact of conductance in pyramidal neurons.. Nat Neurosci.

[33] Markram H, Lubke J, Frotscher M, Roth A, Sakmann B (1997). Physiology and anatomy of synaptic connections between thick tufted pyramidal neurones in the developing rat neocortex.. J Physiol.

[34] Madison DV, Nicoll RA (1984). Control of the repetitive discharge of rat CA 1 pyramidal neurones in vitro.. J Physiol.

[35] Schwindt PC, Spain WJ, Foehring RC, Stafstrom CE, Chubb MC (1988). Multiple potassium conductances and their functions in neurons from cat sensorimotor cortex in vitro.. J Neurophysiol.

[36] Crill WE (1999). Functional implications of dendritic voltage-dependent conductances.. J Physiol Paris.

[37] Stuart G, Sakmann B (1995). Amplification of EPSPs by axosomatic sodium channels in neocortical pyramidal neurons.. Neuron.

[38] Stricker C, Field AC, Redman SJ (1996). Statistical analysis of amplitude fluctuations in EPSCs evoked in rat CA1 pyramidal neurones in vitro.. J Physiol.

[39] Magee JC, Cook EP (2000). Somatic EPSP amplitude is independent of synapse location in hippocampal pyramidal neurons.. Nat Neurosci.

[40] Williams SR, Stuart GJ (2002). Dependence of EPSP efficacy on synapse location in neocortical pyramidal neurons.. Science.

[41] Schwindt PC, Crill WE (1995). Amplification of synaptic current by persistent sodium conductance in apical dendrite of neocortical neurons.. J Neurophysiol.

[42] Magistretti J, Ragsdale DS, Alonso A (1999). Direct demonstration of persistent Na+ channel activity in dendritic processes of mammalian cortical neurones.. J Physiol.

[43] Schiller J, Schiller Y, Stuart G, Sakmann B (1997). Calcium action potentials restricted to distal apical dendrites of rat neocortical pyramidal neurons.. J Physiol.

[44] Yuste R, Gutnick MJ, Saar D, Delaney KR, Tank DW (1994). Ca2+ accumulations in dendrites of neocortical pyramidal neurons: an apical band and evidence for two functional compartments.. Neuron.

[45] Markram H, Sakmann B (1994). Calcium transients in dendrites of neocortical neurons evoked by single subthreshold excitatory postsynaptic potentials via low-voltage-activated calcium channels.. Proc Natl Acad Sci U S A.

[46] Branco T, Hausser M (2011). Synaptic integration gradients in single cortical pyramidal cell dendrites.. Neuron.

[47] Stuart G, Schiller J, Sakmann B (1997). Action potential initiation and propagation in rat neocortical pyramidal neurons.. J Physiol.

[48] Larkum ME, Zhu JJ, Sakmann B (2001). Dendritic mechanisms underlying the coupling of the dendritic with the axonal action potential initiation zone of adult rat layer 5 pyramidal neurons.. J Physiol.

[49] Larkum ME, Zhu JJ (2002). Signaling of layer 1 and whisker-evoked Ca2+ and Na+ action potentials in distal and terminal dendrites of rat neocortical pyramidal neurons in vitro and in vivo.. J Neurosci.

[50] Oakley JC, Schwindt PC, Crill WE (2001). Dendritic calcium spikes in layer 5 pyramidal neurons amplify and limit transmission of ligand-gated dendritic current to soma.. J Neurophysiol.

[51] Schiller J, Major G, Koester HJ, Schiller Y (2000). NMDA spikes in basal dendrites of cortical pyramidal neurons.. Nature.

[52] Hausser M, Mel B (2003). Dendrites: bug or feature?. Curr Opin Neurobiol.

[53] Spruston N, Kath WL (2004). Dendritic arithmetic.. Nat Neurosci.

[54] Larkum ME, Nevian T (2008). Synaptic clustering by dendritic signalling mechanisms.. Curr Opin Neurobiol.

[55] Petreanu L, Mao T, Sternson SM, Svoboda K (2009). The subcellular organization of neocortical excitatory connections.. Nature.

